# Psychometric analysis of the Brazilian-version Kidscreen-27 questionnaire

**DOI:** 10.1186/s12955-021-01824-7

**Published:** 2021-07-27

**Authors:** Pablo Magno da Silveira, Alexsandra da Silva Bandeira, Marcus Vinicius Veber Lopes, Adriano Ferreti Borgatto, Kelly Samara da Silva

**Affiliations:** Research Center in Physical Activity and Health, Federal University of Santa Catarina, Florianópolis, Santa Catarina Brazil

**Keywords:** Adolescence, Cultural sensitivity, Measurement, Quality of life

## Abstract

**Background:**

The objective of this study was to verify the reliability, discriminatory power and construct validity of the Kidscreen-27 questionnaire in Brazilian adolescents.

**Methods:**

Adolescents that participated of the pilot study (210 adolescents; 52.9% boys; 13.7 years old) and of the baseline (816 participants; 52.7% girls; 13.1 years old) of the Movimente Project in 2016/2017 composed the sample of the present study. This project was carried out in six public schools in the city of Florianópolis, Santa Catarina, Brazil. Test–retest reproducibility was assessed by the intraclass correlation coefficient and Gwet coefficient; internal consistency through McDonald's Omega; Hankins' Delta G coefficient verified the scale's discriminatory power and; confirmatory factor analysis to assess construct validity.

**Results:**

Reproducibility values ranged from 0.71 to 0.78 for the dimensions (ICC), and ranged from 0.60 to 0.83 for the items (Gwet). McDonald's Ômega (0.82–0.91) for internal consistency measures. Discriminatory power ranging from 0.94 for the dimension Social Support and Friends to 0.98 for Psychological Well-Being. The factorial loads were > 0.40, except for item 19 (0.36). The fit quality indicators of the model were adequate (X^2^[df] = 1022.89 [311], p < 0.001; RMSEA = 0.053 (0.049–0.087); CFI = 0.988; TLI = 0.987), confirming the five-factor structure originally proposed.

**Conclusions:**

The Brazilian-version Kidscreen-27 achieved good levels of reproducibility, internal consistency, discriminatory power and construct validity. Its use is adequate to measure the health-related quality of life of adolescents in the Brazilian context.

## Introduction

Health-related quality of life (HRQoL) is a perceived and multidimensional health model that describes aspects of well-being and physical, emotional, mental, social and behavioral functions, identified by the individual himself and by others [1]. Its evaluation in children and adolescents is considered an important health indicator, as it is during this period of life that cognitive, physical, psychosocial, emotional and behavioral changes occur and can affect health and well-being [2]. Studies show different associations of HRQoL with biological (sex, age, biological maturation) [2, 3] and behavioral characteristics (physical activity, sedentary behavior, diet, sleep, smoking and alcohol consumption) [2, 4] as well with diseases such as asthma, diabetes, obesity and rare diseases [5].

As a proposal for HRQoL measurement, Kidscreen emerged from a project promoted by a group from the European Union with the participation of thirteen countries with the objective of producing a cross-cultural self-assessment measure for healthy children and adolescents and/or with chronic diseases [6]. The first instrument developed by the group was the version with 52 items (Kidscreen 52), covered in 10 dimensions of HRQoL [1]. Then, in order to provide an adequate tool for large epidemiological and clinical studies, a version with 27items, covering five dimensions, was derived from the 52-item version [1]. Finally, a 10-item version, derived from the 27-item version, was created for them to summarize the dimension scores in a single value-global index [1].

Since its development, all versions have been used in a variety of configurations and study designs in different parts of the world [7–11]. In Brazil, the 52-item version was translated and evaluated for exploratory factor structure and internal consistency [12]. A second study, using the same translated version, evaluated the 27-item version for reproducibility, internal consistency and construct validity through face-to-face interviews [13]. Although adequate validation parameters were observed, the authors highlight that the results found refer to specific context, and that differences sociocultural existing among Brazilian regions should be considered in the use of the instrument. The present study intends to advance in three points: (1) to analyze if the psychometric parameters are kept in a sample of the south region of Brazil; (2) to use the collective interview procedure, and not the face-to-face one [13], considering that in school-based research this procedure is more usual; (3) to perform statistical analysis more appropriate for ordinal categorical data, type of item of the Kidscreen instrument, differently of the another study [13]. Therefore, this study aimed to assess the reliability, discriminatory power and construct validity of the version translated into Brazilian Portuguese language, available on the Kidscreen website.

## Methods

### Design and participants

In order to analyze the objectives of this study, two stages of the “Movimente Program” (www.movimente.ufsc.br) conducted in 2016/2017 in schools in the Florianopolis city in the state of Santa Catarina, southern Brazil [14] were considered: pilot study (May–July 2016) and baseline (March 2017). Characterized as a school-based intervention program, randomized and controlled by conglomerate, the Program was registered in Clinical Trials (NCT02944318) and conducted during a school year (March to December 2017).

In the present study, the sample size calculation was performed in two moments. To estimate reproducibility, the sample size considered an intraclass correlation coefficient (ICC) ≥ 0.20, two applications of the questionnaire, type I error of 5% and type II error of 20% (power of 80%) and an increase 30% for losses and refusals. Following these criteria, a sample of 193 adolescents was necessary. To estimate other parameters (internal consistency, discriminatory power and construct validity), the sample size considered the rate of 20 individuals for each item of the instrument [15]. Considering the 27 items, a total sample of 540 subjects was estimated. For these analyzes, individuals who completed all 27 items were considered.

The pilot study data (all classes from the 7th to the 9th grade of a school), provided elements to evaluate reproducibility. In this phase, the questionnaire was applied in two moments, with an interval of one week. In this case, the literature is not unanimous, but it recommends that this interval should not long (e.g.: 2 months), to avoid possible changes in the phenomenon, nor short (e.g.: 1 day), to avoid that the results are contaminated by the recall effect [16].

The baseline data (all classes from the 7th to the 9th grade of six schools), allowed to examine internal consistency, discriminatory power and construct validity. The adolescents involved in the present study signed the assent form and were authorized by their respective guardians, by signing the consent form. All adolescents, of both sexes, regularly enrolled in the selected schools, attending the first 2 weeks of class (data collection period) were eligible. This study was approved by the Research Ethics Committee of the Federal University of Santa Catarina (CAAE: Protocol Number.: 49462015.0.0000.0121) and by the Florianópolis Municipal Department of Education. The copyright of the Kidscreen instrument used in the research belongs to the Kidscreen Group, under the responsibility of Prof. Ulrike Ravens-Sieberer, MPH. A formal collaboration form was signed to use the instrument.

### Kidscreen 27—Health-Related quality of Life

Kidscreen 27 consists of five dimensions: (1) Physical Well-being (5 items); (2) Psychological Well-being (7 items); (3) Autonomy and Parent relation (7 items); (4) Social Support and Peers (4 items) and (5) School environment (4 items). The 27 items have five response options according to intensity (nothing, little, moderately, very, totally) and frequency (never, rarely, sometimes, often, always), with 1-week recall period. Scores are coded from 1 to 5 and items formulated with negative response categories (1, 9, 10 and 11) are inverted to follow the same direction as positively formulated items [1]. The questionnaire was administered in a similar way at the two collection times (pilot and baseline), being carried out in the classroom during school hours, with completion by the students themselves and with an average duration of 10 min. The assembly of the database was performed through optical reading of the questionnaire, using the SPHYNX software (Sphynx®, Software Solution Incorporation, USA). Possible erasures or errors made by the respondents were checked manually by the team members, who were previously trained to handle the equipment.

### Statistical analysis

To estimate reproducibility, Intraclass Correlation Coefficient (ICC) [17] and Gwet’s coefficient [18] were used in analyzes of dimensions scales and items, respectively. Gwet Agreement Coefficient was proposed as an alternative parameter to assess agreement of categorical variables that overcome known biases related to Cohen’s Kappa [19]. For the ICC, values below 0.50 are considered weak, between 0.50 and 0.75 are moderate, between 0.75 and 0.90 are good and values > 0.90 are excellent indicative [17]. For the Gwet coefficient, values < 0.20 are considered of low agreement, between 0.21 and 0.40 mild, 0.41–0.60 moderate, 0.61–0.80 good and, values > 0, 80 are considered of excellent agreement [20].

To assess internal consistency, the omega coefficient [21], pointed out as an alternative and considered a more sensitive measure than Cronbach's Alpha and appropriate to estimate reliability, mainly of multidimensional instruments where different item scales and factor loads [22]. For the calculation of the ômega coefficient, the Weighted Least Squares Means and Variance estimator (WLSMV) was used as the basis, highlighted in the literature for being more suitable for modeling latent variables with categorical indicators of ordinal level, due to its robustness to modest violations of the underlying normality and good performance in larger sample sizes [23]. Values greater than 0.80–1.00 are considered desirable; indexes between 0.70 and 0.79 are considered recommended and indexes between 0.60 and 0.69 should be accepted for research use only (clinical use is not recommended). Values below 0.60 suggest unreliability of the instrument [21]. For comparison with other studies involving analysis of the psychometric properties of Kidscreen, Cronbach's alpha is presented.

The discriminatory power was determined by Hankins' Delta G, a statistic indicated for scales of dichotomous items, but also for polytomous items, typically five or Likert-type scales [24]. Values between 0 (individuals with the same score, without variability) and 1 (individuals distributed along the scale, with variability) are possible. A score of 0.70 or more is reported as acceptable [24].

Confirmatory factor analysis was used to assess the quality of fit of the model and to compare competing models (construct validity), estimated by the WLSMV. The model adjustment was analyzed considering: chi-square (X^2^[df]) with values of p ≤ 0.05, RMSEA (Root Mean Square Error of Approximation) with values ≤ 0,05 suggest a good fit, CFI (Comparative Fit Index) and TLI (Tucker-Lewis Index) with values > 0.95 indicate proper fit and factorial loads (FL) of the items with values ≥ 0.40 considered acceptable.[25]. All the analyses were conducted on R version 3.6.1 for Windows, using the lavaan package version 0.6–7.

## Results

Of the 251 students who participated in the pilot study, 210 were considered for the reproducibility analyzes, as they filled out the questionnaires at both moments of collection (83.7% response rate). For the baseline sample, 921 students completed the questionnaire and of these, 816 were included in the analyzes because they had complete data on the 27 items of Kidscreen (88.6% response rate). Boys were more present in the pilot study (52.9%) and girls at the baseline (52.7%). The mean age of the pilot study sample was 13.7 ± 1.0 years, while the baseline was 13.1 ± 1.1 years. The mean scores of HRQoL (T-scores: 50 ± 10) ranged from 43.6 points in the dimension Physical Well-being (pilot) to 49.8 points in the dimension Social Support and Peers (baseline), being proportionally higher in the baseline in all five dimensions (Table 1).

**Table 1:** Characteristics of the study samples

Variables	Pilot in 2016	Baseline in 2017	p value
	(n = 210)	(n = 816)	
	n (%)	n (%)	
Sex			0.151*
Boys	111 (52.9)	386 (47.3)	
Girls	99 (47.1)	430 (52.7)	
Age mean (years)^a^	13.7 (1.0)	13.1 (1.1)	< 0.001**
School year			0.009*
7th year	52 (24.8)	291 (35.9)	
8th year	76 (36.2)	258 (31.8)	
9th year	82 (39.0)	262 (32.3)	

HRQoL^b^	Mean (SD)	Mean (SD)	p value
Physical well-being	43.6 (10.3)	44.0 (9.8)	0.693**
Psychological well-being	45.0 (10.6)	45.7 (11.5)	0.409**
Autonomy and parent relation	46.4 (9.9)	46.8 (9.1)	0.558**
Social support and peers	47.6 (10.7)	49.8 (10.3)	0.007**
School environment	46.7 (7.9)	48.6 (8.9)	0.054**

^*^Chi-square test; **Student's t test

^a^Variable with 1 non-response at baseline

^b^T-scores with a mean of 50 and standard deviation of 10

Regarding reproducibility, the intraclass correlation coefficients ranged from 0.71 (School environment) to 0.78 (Physical Well-being), and the Gwet AC_2_ coefficients ranged from 0.60 for item 3 “Have you been physically active (e. g. running, climbing, biking)?” to 0.83 for item 1 "In general, how would you say your health is?", both from the dimension Physical Well-being (Table 2).

**Table 2:** Reproducibility of the Brazilian-version Kidscreen 27 instrument

Dimension/item	n	Coef. (CI 95%)*
*Physical well-being*	208	0.78 (0.72–0.82)
Health condition	207	0.83 (0.79–0.87)
Disposition	209	0.67 (0.61–0.73)
Physical activity	206	0.60 (0.54–0.67)
Run well	210	0.66 (0.60–0.72)
Have energy	209	0.72 (0.67–0.77)
*Psychological well-being*	206	0.75 (0.68–0.80)
Be nice	207	0.66 (0.60–0.71)
Mood	206	0.69 (0.64–0.75)
Have fun	203	0.64 (0.58–0.70)
Sad sense	207	0.69 (0.65–0.75)
Willingness to do nothing	206	0.62 (0.55–0.68)
Felt alone	204	0.65 (0.58–0.71)
Happy as it is	208	0.69 (0.63–0.76)
*Autonomy and parent relation*	204	0.73 (0.66–0.79)
Time for you	208	0.66 (0.60–0.73)
Free time activities	208	0.62 (0.55–0.68)
Time with parents	206	0.62 (0.56–0.68)
Justice treatment by parents	204	0.64 (0.57–0.71)
Parents availability	206	0.69 (0.63–0.74)
Money to make even if friends	205	0.63 (0.45–0.70)
Expenditure money	204	0.61 (0.53–0.68)
*Social support and peers*	205	0.73 (0.65–0.78)
Time with friends	206	0.62 (0.55–0.69)
Fun with friends	203	0.72 (0.66–0.79)
Help among friends	204	0.68 (0.62–0.74)
Trust in friends	203	0.66 (0.59–0.73)
*School environment*	203	0.71 (0.64–0.78)
Happy at school	204	0.73 (0.67–0.78)
Well at school	203	0.81 (0.76–0.86)
Pay attention in class	205	0.71 (0.66–0.76)
Relationship with teachers	206	0.67 (0.61–0.73)

*CI* confidence interval

^*^Parameters were expressed as Intraclass Correlation Coefficients for dimension scores and as Gwet Agreement Coefficients for each item within dimensions

The measure of internal consistency ranged from 0.82 in the dimension School Environment to 0.91 in the dimension Psychological Well-being (Table 3). The floor effects ranged from 0.1 to 0.5%, while the ceiling effects ranged from 2.7 to 16.0%. The scale's discriminatory power ranged from 0.94 in the Social Support and Peers dimension to 0.98 in the Psychological Well-being dimension.

**Table 3:** Internal consistency and discriminatory power of the Brazilian-version Kidscreen 27 instrument (n = 816)

HRQoL dimension	Internal Consistency		% Floor	% Ceiling	Discrimination power
	Alpha	Ômega			
Physical Well-being	0.82	0.84	0.5	2.7	0.97 (0.96–0.97)
Psychological Well-being	0.88	0.91	0.3	5.0	0.98 (0.97–0.98)
Autonomy and Parent relation	0.77	0.84	0.1	3.0	0.97 (0.96–0.97)
Social Support and Peers	0.78	0.84	0.1	16.0	0.94 (0.93–0.94)
School environment	0.76	0.82	0.1	4.2	0.95 (0.94–0.95)

**Fig. 1:**
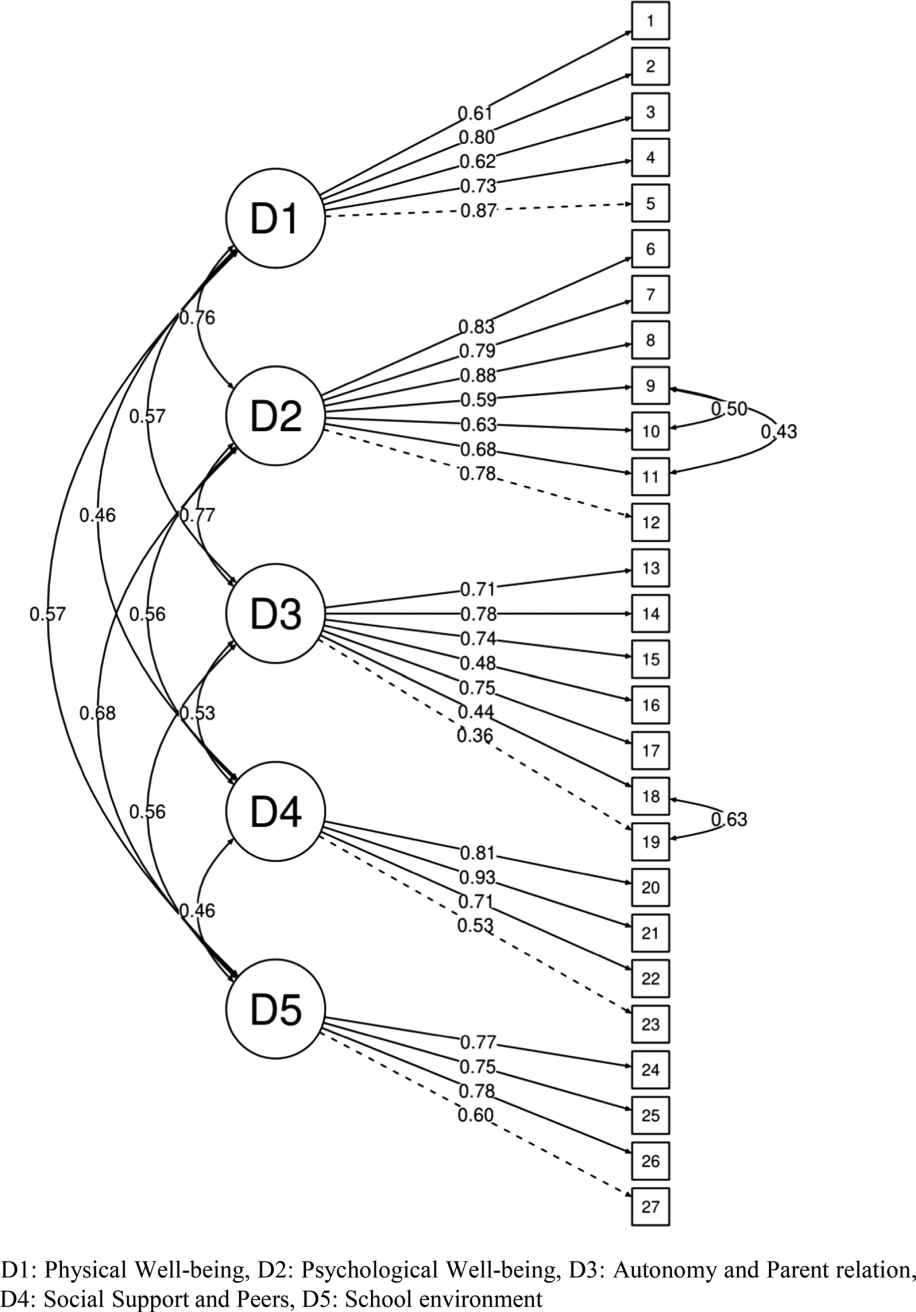
Confirmatory factor analysis of the Brazilian-version Kidscreen 27 instrument

The results showed that the factorial loadings were greater than 0.40, except for item 19 “Do you had enough money for both expenses?” of the “Autonomy and Parent relation” domain (loading = 0.36). Other loadings ranged from 0.43 to item 18 "Do you had enough money to do the same things as your friends?" from the dimension “Autonomy and Parent relation” to 0.93 for item 21 “Have you had fun with your friends?” the “Social Support and Peers” dimension. The correlations between dimensions ranged from 0.46 between the dimensions Social Support and Peers and School Environment to 0.77 between Psychological Well-being and Autonomy and Parent relation (Fig. 1).

The fit quality indicators of the model without error covariances showed that the factorial structure (X^2^[df] = 1936.18 [314], p < 0.001; RMSEA = 0.080 (0.076–0.083); CFI = 0.973; TLI = 0.970). The model was re-specified, adding covariance between the errors of the following items: 18 “Do you had enough money to do the same things as your friends s?” and 19 “Do you had enough money for both expenses?”; 9 "Have you felt sad?" and 10 “Have you felt so bad that you didn’t want to do anything?”; 9 " Have you felt sad?" and 11 “Have you felt lonely?”. This model showed improved fit quality indicators (X^2^[df] = 1022.89 [311], p < 0.001; RMSEA = 0.053 (0.049–0.087); CFI = 0.988; TLI = 0.987).

## Discussion

This study demonstrated that the Kidscreen 27 instrument reached good levels of reproducibility, internal consistency, discriminatory power and construct validity, being adequate to measure the HRQoL of adolescents in the Brazilian context.

A large body of psychometric results from international research involving the Kidscreen-27 instrument allows a direct comparison with our results [1, 7, 9, 13, 26]. As for HRQL scores, previous studies have shown similar quantifications [7, 26] with mean values around 49.8 to 53.9 and higher [9, 13] with values around 52.1–85.7. Explanations for these results may consider issues such as the cultural, socioeconomic and methodological context of the research, factors that can interfere both positively and negatively in the HRQoL scores in the different dimensions of the instrument.

The reproducibility of this study found higher ICC values ranging from 0.71 to 0.78 than the Ravens-Sieberer et al. [26] study with data from 13 European countries and similar to the studies developed by Andersen et al. [7] (ICC: 0.71 a 0.81) and Nezu et al. [8] (ICC: 0.73 a 0.79). Higher values were found in the studies by Quintero et al. [27] (ICC: 0.87 a 0.99), Ng et al. [28] (ICC: 0.78 a 0.86) and Farias Júnior et al. [13] (0.70–0.96). In the present study, the second application of the questionnaire was seven days after the first. Virtually all studies used an interval of seven days or more between applications. Some precautions with the ICC values must be considered, such as the questionnaire application procedure (face-to-face interview, telephone interview, responsible interview (proxy) and self-report) and the age range of the respondents [29]. Specifically regarding administration, Kidscreen was originally developed to be responded through self-reported or parents/guardians [1]. The administration by interview was observed in the study by Farias Júnior et al. [13] and Quintero et al. [27], a question that may explain the slightly higher ICC values, in relation to the values found in this study.

A moderate to good agreement was observed by the Gwet coefficient between the items of the five dimensions, with most items classified as “good agreement” according to the criteria by Landis & Koch [20]. Item 1 “In general, how would you say your health is?”, Was the item with the highest agreement (0.83), followed by item 25 “Have you got on well at school?” (0.81). The other items-maintained values ranging from 0.73 to 0.60. On this issue, the literature has highlighted the frequent use of the Kappa coefficient instead of the Gwet coefficient [30] to analyze the stability of categorical variables. Although Kilen Li Gwet proved in 2002 [19] the superiority of the Gwet coefficient when compared to Cohen's Kappa, few researchers use it as a statistical tool, or are not even aware of its existence [30].

The internal consistency of this study was assessed by McDonald's omega coefficient, an estimator that considers the different standardized factor loads for each item of the instrument. The omega values in the present study ranged from 0.82 for the School Environment dimension to 0.91 for the Psychological Well-being dimension, classified as desirable [21].

The decision by this estimator contradicts most of the validation studies for Kidscreen, which chose to present Cronbach's alpha [31], including by the Kidscreen Group [1]. In view of this, it was decided to present the alpha values as well, as no psychometric study of the Kidscreen instrument was found in the literature involving the omega coefficient for internal consistency analysis. Cronbach's alpha values in the present study ranged from 0.76 for the School environment dimension to 0.82 for the Physical Well-being dimension, values similar to those found by the Kidscreen Group (0.80–0.84) [1], Andersen et al. (0.77 a 0.82) [7] and Shannon et al. (0.65–0.74) [9]. Following Cronbach’s criteria [31], it can be said that the scale has acceptable internal consistency.

The scale's discriminatory power ranged from 0.94 to 0.98. These results are similar to found by the Kidscreen Group (0.81–0.99). This analysis, even recommended [1], has not yet been reported by any study analyzing the psychometric properties of Kidscreen. In this study, we opted for the Hankins Delta G coefficient [24], as it is theoretically more appropriate for the type of items on the Kidscreen, polytomous and graduated response scale.

The CFA supported the five dimensions found in the original study and in other studies [7, 13], demonstrating that the instrument is in accordance with the conceptual and theoretical considerations on the measurement of HRQoL. The loads of the items as well as the correlations between the dimensions were considered good and the multidimensional structure was confirmed. The final model showed an acceptable fit, especially when the modification indices were taken into account. The estimator WLSMV used in this analysis, uses the matrix of polychoric correlations between the items during the factor analysis. Correlations of this nature tend to be, in comparison to Pearson's coefficient, a more consistent estimate of the true linear relationship between variables [23]. Kidscreen validity studies use maximum likelihood as an estimator for exploratory and confirmatory factor analyzes [9, 13].

In the model, three covariance were added, in this order: (i18–i19; i9–i10; i10–i11). Conceptually, the covariance of these items makes sense. Item 18 “Have you had enough money to do the same things as your friends?” and item 19 "Have you had enough money for your expenses?", both of the dimension "Autonomy and Parent relation" are related to financial issues, unlike the other items (13, 14, 15, 16 and 17) that are related to issues of autonomy, relationship with parents and life at home. Although parents are probably at this age, sources of financial support, this seems to be independent of their relationships with them [28].

Other covariance, from item 9 “Have you felt sad?” with item 10 “Have you felt so bad that you didn’t want to do anything?” and item 10 “Have you felt so bad that you didn’t want to do anything?” with item 11 “Have you felt lonely?”, are related to mood and emotion, different from the other items (6, 7, 8 and 12) that are related to psychological characteristics (items 6, 7 and 8) and self-perception (item 12). Andersen et al. [7], when evaluating the psychometric properties of the Norwegian version of Kidscreen 27, draw attention to another detail: items 9, 10 and 11 are formulated “negatively”, different from the other items that are written “positively”, which can influence the contribution of the items to the dimension in question.

In contrast to these findings, two studies confirmed other dimensional structures for Kidscreen in its version with 27 items. Ng et al. [28] evaluated the original proposed five-dimensional model, but the fit was poor: X^2^[df] = 4553.16 [314], p < 0.001; RMSEA = 0.10; CFI = 0.91; TLI = 0.90). After employing the resources of the TRI, through Rasch modeling a seven-dimensional model was identified, presenting a new structure with acceptable fit (X^2^[df] = 2507.43 [303], p < 0.001; RMSEA = 0.07; CFI = 0.95; TLI = 0.95). A study conducted by Quintero et al. [27] performed exploratory factor analysis and confirmed a seven-dimensional version. When excluding item 1 “In general, how would you say your health is?”, Six dimensions remained. At the end, the confirmatory factorial ratified the six dimensions (RMSEA = 0.097; CFI = 0.754; NFI = 0.699; GFI 0.754; AGFI = 0.701).

This study had strengths. Theoretical development and validation of instruments for measuring quality of life in adolescents has become relevant in different contexts in the health field, as it is a recognized way to understand the needs in health services and guide decision making for the allocation of financial resources for health programs [32]. In addition, methodological rigor of processing and analysis of variables was followed as recommended by the Kidscreen Group, which allows external comparisons.

As limitations not tested the convergent validity and sensitivity to change due to the objectives and cross-sectional design of this study. An important fact to be highlighted is that in the version of the Kidscreen 27 instrument, the Self-Perception dimension is represented only by item 12 "Have you been happy with the way you are?" Originally, the construction of the instrument started from a generic proposal, indicated to measure the HRQoL of healthy children and adolescents and/or with chronic diseases. The sample of this study did not count on the participation of adolescents who had physical limitations, which could be interesting to test the psychometric properties.

## Conclusions

Kidscreen 27 is still considered an instrument for measuring generic and cross-cultural HRQoL since its origin. That said, in this assessment process, good levels of reliability were achieved, assessed by test–retest reproducibility and internal consistency, the scale had a great discriminatory power and its five dimensions were confirmed by the construct validity, being indicated to measure the health-related quality of life in Brazilian adolescents. Studies futures should evaluate if items related to mood and emotion should not compose a dimension different from those related to psychological and self-perception characteristics since the direction of the sub-items and the results were quite different.

## Data Availability

The authors declare the transparency of the data and provide the database used in this study.

## Abbreviations

HRQoL: Health related quality of life
ICC: Intraclass correlation coefficient
WLSMV: Weighted least squares means and variance estimator
RMSEA: Root mean square error of approximation
CFI: Comparative Fit Index
TLI: Tucker-Lewis Index
FL: Factorial loads

## References

[1] Ravens-Sieberer U (2006). The Kidscreen questionnaires: quality of life questionnaires for children and adolescents.

[2] Hourani EM, Hammad SM, Shaheen A, Amre HM (2017). Health-related quality of life among Jordanian adolescents. Clin Nurs Res.

[3] Garcia C, Teles J, Barrigas C, Fragoso I (2018). Health-related quality of life of Portuguese children and adolescents according to their biological maturation and volume of physical activity. Qual Life Res.

[4] Muros JJ, Salvador Pérez F, Zurita Ortega F, Gámez Sánchez VM, Knox E (2017). The association between healthy lifestyle behaviors and health-related quality of life among adolescents. J Pediatr (Rio J).

[5] Paz-Lourido B, Negre F, de la Iglesia B, Verger S (2020). Influence of schooling on the health-related quality of life of children with rare diseases. Health Qual Life Outcomes.

[6] Ravens-Sieberer U, Herdman M, Devine J, Otto C, Bullinger M, Rose M (2014). The European KIDSCREEN approach to measure quality of life and well-being in children: development, current application, and future advances. Qual Life Res.

[7] Andersen JR, Natvig GK, Haraldstad K, Skrede T, Aadland E, Resaland GK. Psychometric properties of the Norwegian version of the Kidscreen-27 questionnaire. Health Qual Life Outcomes. 2016;14:58. 10.1186/s12955-016-0460-4 PMC4826483 27062022

[8] Nezu S, Iwasaka H, Saeki K, Obayashi K, Ishizuka R, Goma H (2016). Reliability and validity of Japanese versions of KIDSCREEN-27 and KIDSCREEN-10 questionnaires. Environ Health Prev Med.

[9] Shannon S, Breslin G, Fitzpatrick B, Hanna D, Brennan D (2017). Testing the psychometric properties of Kidscreen-27 with Irish children of low socio-economic status. Qual Life Res.

[10] Berman AH, Liu B, Ullman S, Jadbäck I, Engström K (2016). Children’s quality of life based on the KIDSCREEN-27: child self-report, parent ratings and child-parent agreement in a Swedish random population sample. PLoS ONE.

[11] Silva N, Pereira M, Otto C, Ravens-Sieberer U, Canavarro MC, Bullinger M (2019). Do 8- to 18-year-old children/adolescents with chronic physical health conditions have worse health-related quality of life than their healthy peers? A meta-analysis of studies using the KIDSCREEN questionnaires. Qual Life Res.

[12] Guedes DP, Guedes JERP (2011). Translation, cross-cultural adaptation and psycometric properties of the KIDSCREEN-52 for the Brazilian population. Rev Paul Pediatr Associação Paulista de Pediatria.

[13] Farias Júnior JC de, Loch MR, Neto AJ de L, Sales JM, Ferreira FEL de L. Reproducibility, internal consistency, and construct validity of KIDSCREEN-27 in Brazilian adolescents. Cad Saúde Pública. 2017;33(9):e00131116. 10.1590/0102-311X00131116 28977279

[14] Silva KS, da Silva JA, Barbosa Filho VC, dos Santos PC, da Silveira PM, Lopes MVV (2020). Protocol paper for the Movimente school-based program: a cluster-randomized controlled trial targeting physical activity and sedentary behavior among Brazilian adolescents. Medicine (Baltimore).

[15] Hair J, Black W, Anderson R (2014). Multivariate data analysis.

[16] Kimberlin CL, Winterstein AG (2008). Validity and reliability of measurement instruments used in research. Am J Health-Syst Pharm AJHP Off J Am Soc Health-Syst Pharm.

[17] Koo TK, Li MY (2016). A guideline of selecting and reporting intraclass correlation coefficients for reliability research. J Chiropr Med.

[18] Gwet KL. Handbook of inter-rater reliability, 4th edition: the definitive guide to measuring the extent of agreement among raters. Advanced Analytics, LLC; 2014.

[19] Gwet KL. Kappa statistic is not satisfactory for assessing the extent of agreement between raters. 2002 [cited 2020 Aug 7]. https://agreestat.com/papers/kappa_statistic_is_not_satisfactory.pdf.

[20] Landis JR, Koch GG (1977). The measurement of observer agreement for categorical data. Biometrics.

[21] McDonald RP (1999). Test theory: a unified treatment.

[22] Green SB, Yang Y (2009). Commentary on coefficient alpha: a cautionary tale. Psychometrika.

[23] Flora DB, Curran PJ (2004). An empirical evaluation of alternative methods of estimation for confirmatory factor analysis with ordinal data. Psychol Methods.

[24] Hankins M (2007). Questionnaire discrimination: (re)-introducing coefficient δ. BMC Med Res Methodol.

[25] Kline RB (2011). Principles and practice of structural equation modeling.

[26] Ravens-Sieberer U, Auquier P, Erhart M, Gosch A, Rajmil L, Bruil J (2007). The KIDSCREEN-27 quality of life measure for children and adolescents: psychometric results from a cross-cultural survey in 13 European countries. Qual Life Res Int J Qual Life Asp Treat Care Rehabil.

[27] Quintero CA, Lugo LH, García HI, Sánchez A (2011). Validación del cuestionario KIDSCREEN-27 de calidad de vida relacionada con la salud en niños y adolescentes de Medellín, Colombia. Rev Colomb Psiquiatr.

[28] Ng JYY, Burnett A, Ha AS, Sum KW (2015). Psychometric properties of the Chinese (Cantonese) versions of the KIDSCREEN health-related quality of life questionnaire. Qual Life Res Int J Qual Life Asp Treat Care Rehabil.

[29] Berra S, Ravens-Sieberer U, Erhart M, Tebé C, Bisegger C, Duer W (2007). Methods and representativeness of a European survey in children and adolescents: the KIDSCREEN study. BMC Public Health.

[30] Wongpakaran N, Wongpakaran T, Wedding D, Gwet KL (2013). A comparison of Cohen’s Kappa and Gwet’s AC1 when calculating inter-rater reliability coefficients: a study conducted with personality disorder samples. BMC Med Res Methodol.

[31] Cronbach LJ (1951). Coefficient alpha and the internal structure of tests. Psychometrika.

[32] Robitail S, Ravens-Sieberer U, Simeoni M-C, Rajmil L, Bruil J, Power M (2007). Testing the structural and cross-cultural validity of the KIDSCREEN-27 quality of life questionnaire. Qual Life Res.

